# The Dendritic Cell Synapse: A Life Dedicated to T Cell Activation

**DOI:** 10.3389/fimmu.2016.00070

**Published:** 2016-03-07

**Authors:** Federica Benvenuti

**Affiliations:** ^1^Cellular Immunology, International Centre for Genetic Engineering and Biotechnology, Trieste, Italy

**Keywords:** immune synapse, dendritic cells, actin cytoskeleton, polarized secretion, antigen presentation

## Abstract

T-cell activation within immunological synapses is a complex process whereby different types of signals are transmitted from antigen-presenting cells to T cells. The molecular strategies developed by T cells to interpret and integrate these signals have been systematically dissected in recent years and are now in large part understood. On the other side of the immune synapse, dendritic cells (DCs) participate actively in synapse formation and maintenance by remodeling of membrane receptors and intracellular content. However, the details of such changes have been only partially characterized. The DCs actin cytoskeleton has been one of the first systems to be identified as playing an important role in T-cell priming and some of the underlying mechanisms have been elucidated. Similarly, the DCs microtubule cytoskeleton undergoes major spatial changes during synapse formation that favor polarization of the DCs subcellular space toward the interacting T cell. Recently, we have begun to investigate the trafficking machinery that controls polarized delivery of endosomal vesicles at the DC–T immune synapse with the aim of understanding the functional relevance of polarized secretion of soluble factors during T-cell priming. Here, we will review the current knowledge of events occurring in DCs during synapse formation and discuss the open questions that still remain unanswered.

## Introduction

Once in the lymph node, dendritic cells (DCs) have few hours to transfer the information gathered in the periphery to T cells. Live imaging of the lymph node unveiled that, depending on the inflammatory context and antigen density, T-cell activation can ensue from long-lasting interactions or by collection of signals from subsequent repetitive contacts (1). Recent technologies to visualize recruitment and activation of signaling molecules *in vivo* within individual DC–T contacts is beginning to shed light on the actual functional meaning of various types of intercellular encounters (2, 3). Nevertheless, our present understanding of the subcellular events underlying informational transfer at the synapse and signal decoding by T cells mostly comes from *in vitro* studies. As largely discussed in this topic, sophisticated analysis unveiled the mechanism by which the antigen-presenting cell (APC)-derived flow of information is turned into fine tuned T-cell activation. Less is known, instead, about the events that control efficient delivery of antigen-derived signals to T cells. Among APCs, DCs are uniquely potent in their ability to launch adaptive responses. They are composed of a complex network of different subsets that differentially control T-cell functions. The classical CD8α^+^ with the ontogenetically related tissue resident CD103^+^ cells and the Cd11b subsets share some general principles, i.e., the ability to migrate from tissue to regional lymph nodes charged with antigens, but are specialized in the activation of CD8^+^ and CD4^+^ T lymphocytes, respectively (4). Yet, this paradigm is still evolving and these distinctions may turn out to be less strict.

In the following paragraphs, the events that takes place on the DCs side of the immune synapse (DC-IS) refer mostly to studies conducted using DCs models, i.e., cells differentiated from monocytes in the human system or from bone marrow precursors in the mouse system and unfortunately do not yet take into account such complexity. Still, common themes have emerged that will certainly be translated into more physiological cell types in the near future.

## Surface Receptors

To simplify and categorize events occurring at the DC-IS, it is useful to proceed from the surface inward to the cytoplasm. Remodeling of the plasma membrane architecture and redistribution of the surface receptors have been documented in DCs from the earliest instance upon T-cell contact and even before the two cell bodies become adherent. Some conserved mechanisms to support intercellular interactions described in the neuronal synapse operate in DCs and are essential for contact formation, including semaphorins, plexins, and neuropilins (5). Plexin-1A, a receptor for class-3 semaphorins is highly expressed in mature DCs, cluster at the IS, and controls T-cell priming by regulating cytoskeletal remodeling, possibly *via* Rho activation (6, 7). A second receptor for semaphorins, neuropilin-1, is expressed on both sides of the synapse and can control DC–T encounter during priming and during instruction of regulatory T cells (8, 9). The way DCs interact with T cells is peculiar in terms of global geometry and microdomains organization, as compared with the synapse between B cells and T cells. Unlike B cells, DCs possess a highly dynamic membrane with projections such as veils and ruffles that are increased during the process of maturation (10, 11) and further modify when an approaching T cell is sensed by chemokine receptors (12, 13). In human DCs, a peculiar microvilli-like structure was shown to be the preferential site of association with T cells leading to multiple aggregates of TCR/CD28 signaling complexes in T cells, as opposed to the concentrically structured immune synapse formed by B cells (14). A multifocal synapse is frequently formed also in murine T cells interacting with DCs, further strengthening that antigen presentation by DCs is more dynamic than by B cell (14, 15). Mirroring TCR clustering, MHC class-II and costimulatory molecules such as CD86 are recruited at the DC contact area very early during initial DC–T scanning. This recruitment is driven by ICAM-1/3 interactions with LFA-1 *via*, a mechanism that depends on an intact actin cytoskeleton and the Vav1/Rac1 effectors (16). Delivery of MHC class-II at the plasma membrane depends on long tubules of late endosomal origin that contain MHC–peptide complexes and move directionally toward the contact zone. This process is conserved in murine and human DCs both during antigen presentation and cross-presentation (17, 18) and ensures continuous fueling of TCR ligands in the IS region. Other costimulatory molecules undergo spatial reorganization on the APC. CD40 clusters at the synapse in B cells during delivery of T-cell help and it is stored in intracellular vesicles and rapidly released upon contact with an allogeneic CD4 T cells in rat lymph DCs (19, 20). Mirroring CD40 clustering, CD40L in T cells is recruited at the IS with human DCs and this polarization is important for DCs–T cross-talk and T-cell induced DCs maturation as it triggers IL-12 production by DCs (21). T-cell-induced DC maturation provides an important demonstration of the mutual exchange that occurs between the two cells [reviewed in Ref. (22)]. A further costimulatory molecule CD70, a TNF-family member receptor with critical roles in T-cell priming, is concentrated at the DC-IS. Interestingly, CD70 is found in intracellular compartments that overlap with class-II positive vesicles, and this localization depends on the Invariant chain (Ii), suggesting a shared control of antigen peptides and costimulatory molecules transport to the synaptic area (23, 24). Similarly, ICAM-1 is recycled at the interaction zone through recycling compartments where it intersects MHC class-II molecules (25). ICAM-1 mobility on the DCs membrane is a critical factor controlling activation of T cells. Besides being the main adhesive force to stabilize the interaction, LFA-1 is important to modulate signaling from the TCR. Binding of LFA-1 to its ligand, in fact, enhances signaling for key TCR signal transduction molecules, such as PI3K, PLC-γ, MAP kinases, and SLP-76, reinforcing IL-2 production and T-cell proliferation (26–29). A recent study revealed that cytoskeletal remodeling during DC maturation causes a decrease in the lateral mobility of ICAM-1 in the membrane. Laterally confined ICAM-1 imposes forces on the interacting LFA-1 that promote ligand-dependent activation and increase T-cell priming (30).

## Signaling Molecules

Underneath the plasma membrane and concomitantly to redistribution of membrane receptors, several events take place in the cytoplasm of DCs, suggesting that the DC-IS is an active signaling zone. Spinophilin is a PDZ domain-containing protein that is highly expressed in the dendritic spines of neurons where it controls interactions with the underlying cytoskeleton and with membrane trafficking proteins. Spinophilin was shown to be expressed in DCs and to cluster at the DC membrane in antigen-specific conjugates, and its depletion caused a strong inhibition of T-cell priming (31). This study did not address the consequences of spinophilin loss in terms of cytoskeletal proteins distribution and overall cell symmetry. It is likely that spinophilin participates in the network of PDZ domain-containing proteins regulating cell polarity, indicating the importance of asymmetric distribution of functional subdomains for antigen presentation.

Durable presentation of antigens to T cells, either *via* long-lasting contacts or cumulative interactions, is essential for efficient priming of T cells in lymph nodes (1). The life span of DCs is short, and it is therefore essential to control their fitness when they come to accomplish their ultimate task in the lymph node. Intriguingly, it was demonstrated that synapse formation provides antiapoptotic signals to DCs. Formation of the synapse induces recruitment and activation of the antiapoptotic protein Akt at the DC-IS followed by activation of prosurvival signals, including inhibition of FOXO and activation of NF-κB [(32) and reviewed in Ref. (33)]. Given the extensive remodeling of actin and microtubule cytoskeleton in DCs engaged in synapses (see below), it is likely to expect that other large signaling complexes become selectively recruited and activated at the site of interaction. However, the membrane receptors primarily involved, the composition of these signaling platforms, and the hierarchy of their assembly still remains to be elucidated.

## The Actin Cytoskeleton

All synaptic events, from intercellular contact formation and adhesion to signal transduction, are orchestrated by the actin and microtubules cytoskeleton, their upstream regulators and downstream effectors in a continuous feedback loop.

Soon after the discovery of the supramolecular activation cluster in T cells, researchers discovered that DCs actively participate in synapse formation by polarizing the actin cytoskeleton, unlike B cells. The causal link was strong as F-actin pharmacological inhibitors inhibited the T-cell priming capacity of DCs (34). Actin remodeling is controlled by a set of proteins that promote actin nucleation and polymerization, in turn controlled by Rho GTPases. Studies to decipher the molecular pathways involved in the control of actin remodeling in DCs showed, at first, an involvement of the actin-bundling protein, fascin. This protein is highly expressed selectively in DCs, upregulated during maturation, and necessary for DCs full antigen-presenting function (35). Few years later, we found that one essential mediators of actin remodeling during synapse formation in DCs is the small GTPases, Rac. Genetic deletion of Rac1 and 2 rendered DCs unable to establish productive interaction with T cells, leading to extremely inefficient T-cell activation (36). Two main classes of actin nucleating factors, formins and WASp family, involved, respectively, in the formation of long actin polymer elongation and branched actin networks were both shown to play a role in DCs migration and interaction with T cells. Deletion of mDia, a formin family member, inhibits DCs migration to LNs and decreases the capacity to establish long contacts with T cells and to induce their activation (37). WASp, expressed selectively in hematopoietic cells and target of mutations in Wiskott–Aldrich syndrome (38), controls various DCs functions, including the capacity to form stable interactions with T cells and the stability and structure of the synapse. *In vivo* two-photon microscopy analysis in the 3D environment of the lymph node showed decreased contact time between WASp null DCs and CD8^+^ T cells. Closer inspection *in vitro* revealed a defect in accomplishing the complete cycle of movements required to establish a full contact. As a consequence, WASp null conventional DCs often undergo repetitive contacts of short duration, resulting in decreased T-cell proliferation and IFN-γ production (39). Similarly, CD4 T cells are inefficiently primed by WASp null DCs *in vivo* and *in vitro* (40). In this last work, Ca^2+^ signaling and clustering of signaling molecules were shown to be decreased in T cells interacting with mutant cells, indicating that failure to sustain proximal events represents an important contribution to the overall immunodeficiency in WAS. Recently, a novel cell-free system to mimic the T-cell surface was used to address the role of WASp at the DC-IS with increased resolution. The results show that a spatially organized structure containing MHC-II, ICAM-1, and actin forms at the DC-IS, and it is stabilized in a WASp- and Arp2/3-dependent manner (41). It would be interesting to investigate whether WASp is involved in controlling the lateral mobility of ICAM-1, a parameter recently emerged to be critically during antigen presentation by DCs (30). Interestingly, the function of WASp goes beyond the control of motility and signaling at the IS as it also acts in tuning innate responses by toll-like receptors in plasmacytoid DCs (42).

**Figure 1:**
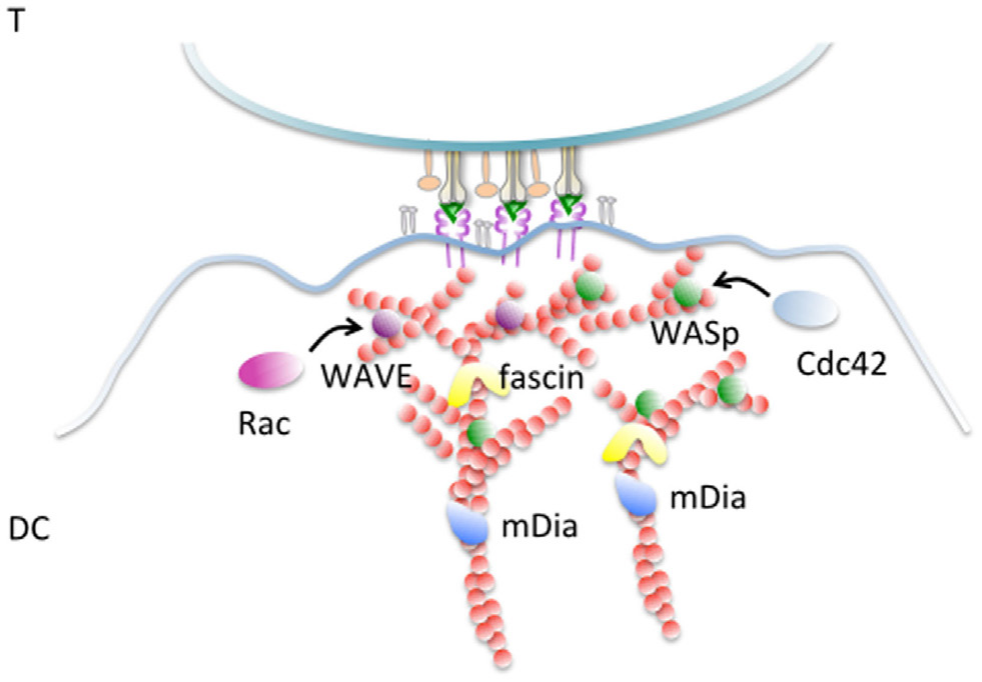
**Actin remodeling at the DC-IS**. F-actin filaments become polarized and cluster enriched at the junction with an antigen-specific T cells. Fascin, an actin-bundling protein implicated in extension of membrane protrusion and development of dendrites is enriched in this area and controls the capacity of DCs to activate T cells. The Rho GTPase Rac is required for synapse formation and T-cell activation, likely *via* WAVE-mediated actin remodeling. WASp is a further activator of the Arp2/3 complex that is essential to support the antigen-presenting activities of DCs by promoting actin branching, thereby stabilizing the synaptic structure. WASp activation is mediated by cdc42 that is also found enriched at the DC-IS. The formin family mDia, involved in elongation of long actin filaments is important to establish and stabilize DC–T contacts and to support T-cell activation.

The role of actin and actin regulatory proteins involved in synapse formation in DCs is summarized in Figure 1. Despite these insights, the triggers and the players of actin remodeling during synapse formation in DCs are not yet understood in sufficient details. As shown in a recent elegant study, DCs switch between different actin nucleating machineries during the process of migration and antigen uptake (43). What type of actin machinery prevails during antigen presentation at the IS and how the switch to this third DCs’ specific function is achieved remains an interesting open question.

## Polarity and Secretion on the APC Side of the Immune Synapse

Polarity is a highly conserved mechanism to spatially organize different functions within a cell, common to many processes, including the regulation of immune cells functions. The importance of polarity is well characterized in T cells where centrosome polarization in CTLs and T-helper cells is a fairly well-understood mechanism to optimize release of cytotoxic granules and cytokines, respectively (44–46). In B cells, centrosome polarization at the synapse is a mechanism to orient the secretion of lysosomes and to promote the extraction of immobilized BCR ligands (47). B cells were shown to polarize also in the context of a B–T cell synapse with both the centrosome and the Golgi apparatus reoriented toward the interaction zone (48).

**Figure 2:**
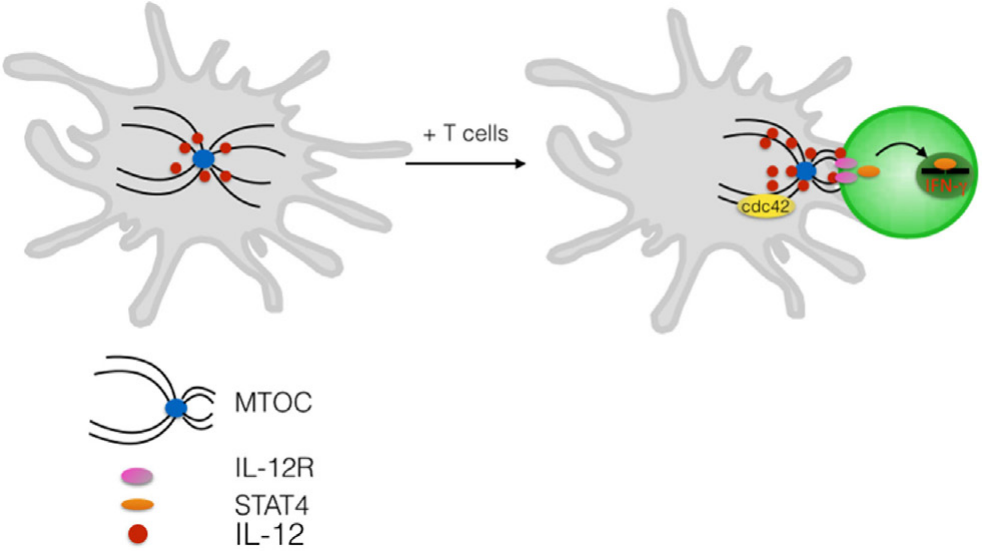
**Polarized delivery of cytokine at the DC-IS**. Upon contact with antigen-specific T cells DCs reorient the centrosome toward the interacting T cell. Polarization depends on the Rho GTPase cdc42. Newly synthesized IL-12 is contained in intracellular vesicles that cluster in the Golgi area and are readily redistribute at the IS upon antigen-specific contact formation. Release of IL-12 in the synaptic cleft induces activation of STAT4 and triggering of IFN-γ in the interacting T cell.

Many evidences exist that cell polarity and polarized membrane trafficking establishes also in DCs upon formation of antigen-specific conjugates. For instance, tubules of MHC class-II emanating in the direction of the interacting T cell indicate that remodeling of membrane trafficking is induced by antigen-specific contacts (17). The ability to polarize the tubules is acquired upon signals from toll-like receptors and depends on microtubules integrity (49, 50). DCs contacting NK cells were shown to polarize cytokines in the synaptic cleft, further suggesting directional vesicular transport (51, 52). Indeed, it was formally demonstrated that DC, following induction by a microbial maturation stimuli, polarizes the microtubule-organizing center (MTOC) toward the DC–T interface (53). This process depends on cdc42 as genetic deletion of this key polarity protein hampers the ability of DCs to reposition the centrosome underneath the synaptic membrane. Cdc42 is indeed highly enriched at the contact zone in DCs indicating its possible role as a WASp activator as well. Vesicles carrying newly synthesized interleukin 12, a key mediator of T-cell priming, clustered around the Golgi and were rapidly repositioned at the DC-IS, resulting in polarized secretion and local induction of IL-12-dependent signaling in T cells (53) (Figure 2). Thus, similarly to what have been shown in T cells, polarized transport of soluble mediators is a fundamental process that contributes to the antigen-presenting properties of DCs as ablation of cdc42-mediated polarization inhibits T-cell proliferation (53). The notion that the so-called signal 3 (proinflammatory cytokines) can be locally coupled to antigenic and costimulatory signals, and it can be delivered in *cis* may have important consequences for the fate imprinted to different T cells that interact sequentially with the same APC.

These findings raise the question of the link between microtubules and membrane trafficking proteins that support the directional transport of mediators en route to exocytosis. In macrophages and in T cells, members of the SNARE and Rab family were shown to control selectively directional secretion of various cytokines (44, 54). In DCs, we have recently discovered that the SNARE VAMP-7 plays a key role in the intracellular trafficking and secretion of newly synthesized IL-12. VAMP-7 not only controls the multidirectional transport of the cytokine toward the plasma membrane but, most importantly, it is absolutely required for clustering and secretion of IL-12 at the DC-IS (55). These findings reveal an usual transport route *via* late endosomes and represent the first insight into the molecular mechanism that orchestrate trafficking and secretion of soluble factors in DCs.

## Conclusion

Both synaptic partners contribute to the successful outcome of the intercellular interaction between an activated DCs and a naive T cell, i.e., T-cell activation.

It is becoming obvious that a complex cross-talk between the two cells exists and that understanding T-cell activation cannot do without a deep understanding of the DCs synapse. Further efforts are envisaged in at least two directions. The first is the systematic dissection of the receptors and the downstream effectors operating sequentially in DCs and their functional significance. This goal will be achieved, thanks to the recent development of cell-free system to reconstitute the T-cell membrane. Second, to increase physiological relevance, future studies should take into account the complexity of the DCs system, which is composed of various DCs subsets that are most likely equipped with different tools to transmit information to their partners.

## Author Contributions

The author confirms being the sole contributor of this work and approved it for publication.

## Conflict of Interest Statement

The author declares that the research was conducted in the absence of any commercial or financial relationships that could be construed as a potential conflict of interest.
